# Rising from the pandemic ashes: Reflections on burnout and resiliency from the infection prevention and antimicrobial stewardship workforce

**DOI:** 10.1017/ash.2022.240

**Published:** 2022-06-22

**Authors:** Priya Nori, Michael P. Stevens, Payal K. Patel

**Affiliations:** 1 Division of Infectious Diseases, Department of Medicine, Montefiore Health System, Albert Einstein College of Medicine, Bronx, New York; 2 Division of Infectious Diseases, Veterans’ Affairs (VA) Ann Arbor Healthcare System and University of Michigan School of Medicine, Ann Arbor, Michigan; 3 Division of Infectious Diseases, Virginia Commonwealth School of Medicine, Richmond, Virginia

## Abstract

Hospital epidemiologists, infection preventionists, and antimicrobial stewards are integral to the pandemic workforce. However, regardless of pandemic surge or postsurge conditions, their workload remains high due to constant vigilance for new variants, emerging data, and evolving public health guidance. We describe the factors that have led to burnout and suggest strategies to enhance resilience.

Throughout the current coronavirus 2019 (COVID-19) pandemic, healthcare workers have faced increasing workloads. Novel variants, emerging data, and evolving public health guidance comprise additional burdens to the hospital epidemiology and antimicrobial stewardship workforce. Here, we describe burnout factors for the infection prevention and antimicrobial stewardship community and we suggest strategies to enhance resilience (Fig. 1).

**Figure 1.**
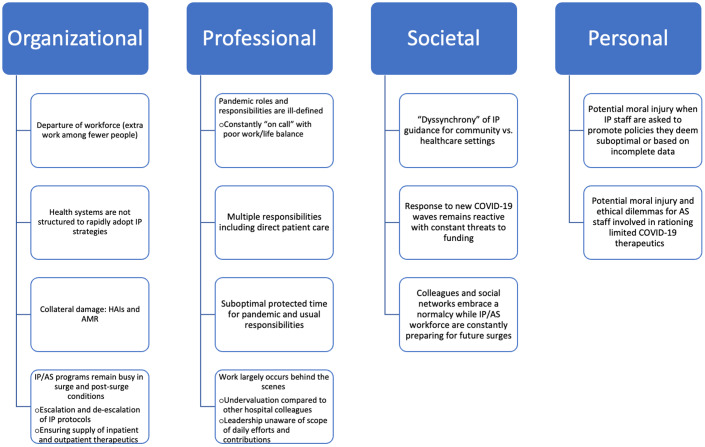
Complex factors contributing to IP/AS burnout

## Staffing

Perennially understaffed, infection prevention (IP) and antimicrobial stewardship (AS) responsibilities have expanded dramatically during the current COVID-19 pandemic.^1^ “The Great Healthcare Resignation” has further strained institutions and has imposed further challenges on existing IP and AS duties.^2^

Furthermore, women account for 41% of infectious disease (ID) physicians, 52% of trainees, and ∼96% of infection preventionists.^3,4^ Women are disproportionately impacted by pandemic-related domestic responsibilities and have been less academically productive.^5^

## Healthcare versus society

“Dyssynchrony” between IP guidance for community versus healthcare settings has contributed to confusion and tension with hospital staff and leadership. Community COVID-19 metrics have been recalibrated to facilitate “pandemic tolerance” and incentives to continue optimal preventive behaviors have diminished. Recurring waves of new severe acute respiratory coronavirus virus 2 (SARS-CoV-2) variants render the work of IP and AS professionals insurmountable. Constant adaptation to variants with shifting antiviral effectiveness requires frequent revision and dissemination of treatment protocols as well as maintenance of a complex and changing drug stock.^6^ The burnout milieu is compounded by reactive federal responses to new surges and constant threats to pandemic funding.

## Competing priorities of the individual versus organization

Throughout the pandemic, IP and AS programs have focused on managing crises in real time, but reliance on leadership to disseminate important messaging may result in delays. Organizations must maintain financial viability and favorable community relationships while IP programs aim to curb nosocomial outbreaks and exposures. Since 2020, fewer institutions have suspended elective procedures during major surges.^7^ Financial losses and staff departures create setbacks to building new programs or implementing IP and AS innovations. Competing priorities may result in IP and AS leaders being excluded from policy discussions despite their applicable expertise.

## Isolation within the healthcare setting

Periods of declining COVID-19 rates signal a respite for many in healthcare, but IP and AS staff remain busy. They must de-escalate protocols, prepare for future surges, and confront the aftermath of healthcare-associated infections and antimicrobial resistance.^8^

## Imposter syndrome

Imposter syndrome describes high-achieving individuals who, despite objective success, fail to internalize accomplishments and have persistent self-doubt. It is associated with fear of failure, low self-esteem, isolation, and is comorbid with depression and anxiety.^9^

Globally, IP and AS professionals were thrust into pandemic leadership roles, often without time to develop requisite skills. Academically, many report “nothing to show for” their often Herculean efforts due to little time for scholarship. For others, rapid acceleration in visibility led to a mismatch of regional or national repute compared to local valuation and compensation within their organizations.

## Psychological distress and moral injury

Early on, IP and public health professionals experienced significant psychological distress from rapidly issuing uncertain policies that affect thousands of people, while being subject to vitriol, threats, and moral injury.^10,11^ At the same time, AS programs have faced hardship when working to establish protocols for novel medications that are supported by poor data. During the onset of the massive surge of the SARS-CoV-2 ο (o)micron variant, effective antiviral agents were scarce but demand was extremely high.^12,13^ Despite significantly improved national reserves, utilization in poor, disconnected communities remains grossly inadequate,^14,15^ compounding the moral injury experienced by providers.

## Diminishing returns

IP and AS expertise is valued by organizations, though often not rewarded. In contrast to earlier phases of the pandemic, demand for IP advice remains high but is increasingly not followed. IP and AS practitioners continue to spend copious uncompensated time addressing personal or patient-specific COVID-19 questions from colleagues, which is not limited to business hours. In our opinion, an organizationally imposed “citizenship tax”^16^ may disproportionately impact IP and AS professionals, though further study is needed.

Likewise, time spent providing infectious diseases expertise to media outlets, while garnering prestige for organizations, is uncompensated, has ill-defined benefits in terms of academic promotion, and may leave these professionals vulnerable to harassment.

## Resiliency specific to epidemiology, IP, and AS communities

Goff et al^17^ describe numerous examples of global resiliency exhibited by AS programs such as leveraging technology and remotely engaging in collaborative research. Although we lack expertise in managing healthcare worker burnout specifically, we suggest the following practical solutions:

1. **Say “no”**: Exhaustion and lack of respect for boundaries should not be normalized. We must differentiate between tasks which contribute to achievement of professional goals (eg, improving patient safety, acquiring executive skills, scholarship, and academic promotion) from those that distract from goals and deliverables.
2. **Say “yes”**: All IP and AS practitioners should seek institutionally sponsored leadership courses or professional coaches, which will benefit the individual, organization, and patients. Time spent on hobbies can also boost workplace performance by building creativity and perspective.^18^
3. **Destigmatize the mental health crisis**: Institutions must recognize the insults specific to the IP, AS, and public health communities, provide tools to “de-brief” and confront traumatic situations, ensure protected time and financial support for mental health treatment when necessary, and provide ongoing support.^19^
4. **Collaborate on our terms**: The pandemic has afforded multiple opportunities to collaborate in medical education, simulation training,^20^ international knowledge sharing,^21,22^ opinion pieces,^23^ and primary research projects. These partnerships have contributed significantly to our understanding of the pandemic and have lain the foundation for future collaborations.
5. **Engage with peer-learning platforms**: Local IP and AS work can be isolating. Recurring webinars, such as the Society for Healthcare Epidemiology of America (SHEA) Town Halls or Centers for Disease Control and Prevention (CDC)/Infectious Diseases Society of America (IDSA) Clinician Calls, keep us informed and allow us to share lessons learned and to engage virtually with colleagues across the world.^24,25^
6. **Embrace external opportunities**: Some IP and AS practitioners have embraced consultative work for corporations, sports teams, local governments, etc. This work can increase personal and professional value and societal engagement during an isolating time.
7. **Adopt carefully curated media and social media**: Some have embraced media platforms and/or social media, which if kept professional, can spark thought-provoking discussions and “crowd-sourced” research collaborations. These outlets can help cultivate a larger professional identity outside our organizations.
8. **Attend in-person professional events**: Althgough virtual conferences are affordable and convenient, professional society meetings have resumed safely and successfully, and have revived personal connections and professional synergies.
9. **Get “back to business”**: As societal focus on COVID-19 recedes, reinvigorating surveillance efforts and expanding our knowledge of HAI/AMR transmission, prevention, and management is a worthwhile and sustaining investment of our energy and resources.
10. **Sustain the IP and AS workforces**: The long-term success of our communities depends on the expansion and maintenance of the workforce through financial investments in training, protected effort, and recruitment. Hence, professional societies have intensified advocacy for increased federal funding for IP, AS, and public health programs.^26^

In conclusion, we have adapted to multiple COVID-19 surges and have developed coping strategies centered in personal and professional connections across the globe.^15^ We should intensify efforts to collaborate virtually or in person, pursue professional activities that hold value, and take advantage of pandemic “respites” to invest in the activities that renew us and bolster resilience.
